# Supplementary material for: Prospective Screening of Cancer Syndromes in Patients with Mesenchymal Tumors
Source: Cancers (Basel). 2024 Nov 13;16(22):3816. doi: 10.3390/cancers16223816 (PMC11592761; doi:10.3390/cancers16223816)
Supplement: Supplementary file 1 [file cancers-16-03816-s001.zip › cancers-3276678-supplementary.pdf]

Supplementary Table S1

AAGAB	*CD79A*	*ERCC5*	*HDAC4*	*MALT1*	*NTRK1*	*RNASEL*	*STAT3*
ABCA5	*CD79B*	*ERCC6*	*HFE*	*MAP2K1*	*NUMA1*	*RNF139*	*STK11*
ABCB11	*CD81*	*ESCO2*	*HIST1H3B*	*MAP2K2*	*NUP214*	*RNF43*	*STK4*
ABCC6	*CD96*	*ETV6*	*HLA-DRB1*	*MAP2K4*	*OCA2*	*RNF6*	*STS*
ABL1	*CDC73*	*EVC*	*HMBS*	*MAP3K1*	*OCRL*	*RPGRIP1L*	*SUFU*
ACAN	*CDH1*	*EVC2*	*HMGA2*	*MAP3K8*	*OFD1*	*RPL10*	*TAF15*
ACD	*CDH23*	*EWSR1*	*HNF1A*	*MAPK1*	*OGG1*	*RPL11*	*TAL1*
ACTB	*CDK4*	*EXO1*	*HNF1B*	*MAPRE2*	*PALB2*	*RPL15*	*TAL2*
ACTG2	*CDKN1A*	*EXT1*	*HNF4A*	*MAX*	*PALLD*	*RPL18*	*TBX18*
ACVR1B	*CDKN1B*	*EXT2*	*HOXB13*	*MBTPS2*	*PARN*	*RPL26*	*TCF3*
ACVRL1	*CDKN1C*	*EYA1*	*HOXD13*	*MC1R*	*PAX3*	*RPL27*	*TCF4*
ADA	*CDKN2A*	*EZH2*	*HPGD*	*MCM4*	*PAX5*	*RPL35*	*TCOF1*
ADAMTS3	*CDKN2B*	*FAH*	*HPS1*	*MDC1*	*PAX6*	*RPL35A*	*TCTN3*
AGGF1	*CDKN2C*	*FAM111B*	*HPS3*	*MDH2*	*PAX7*	*RPL5*	*TEK*
AIP	*CDKN2D*	*FAM175A*	*HPS4*	*MDM2*	*PBRM1*	*RPS10*	*TERC*
AKT1	*CEBPA*	*FAM20A*	*HPS5*	*MDM4*	*PDCD10*	*RPS14*	*TERF1*
ALK	*CEP57*	*FAM20C*	*HPS6*	*MED12*	*PDE6D*	*RPS17*	*TERF2IP*
ALX3	*CHD7*	*FAN1*	*HRAS*	*MEN1*	*PDGFB*	*RPS19*	*TERT*
ALX4	*CHEK1*	*FANCA*	*HSPG2*	*MET*	*PDGFRA*	*RPS20*	*TET2*
AMER1	*CHEK2*	*FANCB*	*HUS1*	*MFN2*	*PDGFRB*	*RPS24*	*TFAP2A*
ANKRD26	*CHRNG*	*FANCC*	*ICOS*	*MGAT2*	*PDGFRL*	*RPS26*	*TFE3*
ANTXR1	*CIC*	*FANCD2*	*IDH1*	*MGMT*	*PHF21A*	*RPS27*	*TG*
ANTXR2	*COL11A2*	*FANCE*	*IDH2*	*MINPP1*	*PHF6*	*RPS28*	*TGFBR2*
AP2S1	*COL14A1*	*FANCF*	*IGF2*	*MITF*	*PHOX2B*	*RPS29*	*THPO*
AP3B1	*COL18A1*	*FANCG*	*IGF2R*	*MLF1*	*PICALM*	*RPS7*	*TIMELESS*
APC	*COL1A1*	*FANCI*	*IGHM*	*MLH1*	*PIEZO2*	*RRAS*	*TINF2*
APC2	*COL2A1*	*FANCL*	*IGLL1*	*MLH3*	*PIGA*	*RSPO1*	*TJP2*
AR	*COL4A5*	*FANCM*	*IKBKG*	*MLLT10*	*PIGL*	*RTEL1*	*TMC6*
ARHGAP26	*COL4A6*	*FAS*	*IKZF1*	*MLLT11*	*PIK3CA*	*RUNX1*	*TMC8*
ARID1A	*COL7A1*	*FASLG*	*IL12A*	*MMEL1*	*PIK3R1*	*SAMD9*	*TMEM127*
ARID1B	*COMP*	*FAT4*	*IL12RB1*	*MMP1*	*PLAG1*	*SAMD9L*	*TMEM216*
ARID2	*CPLX1*	*FBXW7*	*IL2RG*	*MN1*	*PLCD1*	*SBDS*	*TMEM67*
ARMC5	*CR2*	*FCN3*	*IL7R*	*MNX1*	*PMS1*	*SCN4A*	*TNFAIP3*
ARSA	*CREB1*	*FDPS*	*ING1*	*MPL*	*PMS2*	*SCN9A*	*TNFRSF10B*
ASCC1	*CREBBP*	*FERMT1*	*INPP5E*	*MRE11*	*PMVK*	*SDHA*	*TNFRSF13B*
ASCL1	*CRKL*	*FGF3*	*INTU*	*MRE11A*	*PNP*	*SDHAF2*	*TNFRSF13C*
ASPSCR1	*CRLF2*	*FGFR1*	*IRF1*	*MS4A1*	*POLA1*	*SDHB*	*TNFRSF1B*
ASXL1	*CSF1R*	*FGFR2*	*IRF5*	*MSH2*	*POLD1*	*SDHC*	*TNFRSF4*
ATM	*CSF3R*	*FGFR3*	*ITK*	*MSH3*	*POLE*	*SDHD*	*TNFSF12*
ATP7A	*CTBP1*	*FGFRL1*	*JAG1*	*MSH6*	*POLH*	*SEC23A*	*TNFSF15*
ATP7B	*CTC1*	*FH*	*JAGN1*	*MSR1*	*POLR1C*	*SEC23B*	*TNPO3*
ATR	*CTHRC1*	*FIBP*	*JAK1*	*MST1*	*POLR1D*	*SEMA3C*	*TOP2A*

ATRX	*CTLA4*	*FLCN*	*JAK2*	*MT-CO1*	*POLR2A*	*SEMA3D*	*TOP3A*
AXIN1	*CTNNA1*	*FLNA*	*JAK3*	*MT-CO2*	*PORCN*	*SERPINA1*	*TOPBP1*
AXIN2	*CTNNB1*	*FLT3*	*KARS*	*MT-CO3*	*POT1*	*SETBP1*	*TP53*
B2M	*CTR9*	*FLT4*	*KAT6B*	*MT-ND1*	*POU2AF1*	*SETD2*	*TP53BP1*
B3GALT6	*CTSA*	*FN1*	*KCNAB2*	*MT-ND4*	*POU6F2*	*SF3B1*	*TP63*
BABAM1	*CTSC*	*FOXC2*	*KCNE3*	*MT-ND5*	*PPM1D*	*SFTPA2*	*TRAF7*
BAP1	*CXCR4*	*FOXE1*	*KCNH1*	*MT-ND6*	*PPP2R1A*	*SFTPC*	*TREM2*
BARD1	*CYLD*	*FOXI1*	*KCNJ10*	*MT-TL1*	*PPP2R1B*	*SH2B3*	*TREX1*
BCCIP	*CYP11B1*	*FOXL2*	*KCNQ1*	*MT-TS1*	*PRCC*	*SH2D1A*	*TRIM28*
BCL10	*CYP11B2*	*FOXO1*	*KCNQ1OT1*	*MT-TS2*	*PRDM1*	*SH3GL1*	*TRIM37*
BCL2	*CYP26C1*	*FOXP1*	*KDM5C*	*MTAP*	*PRDM16*	*SHH*	*TRIP13*
BCL6	*CYSLTR2*	*FUBP1*	*KDM6A*	*MTM1*	*PRF1*	*SHOC2*	*TRPS1*
BCOR	*DAXX*	*FUZ*	*KDR*	*MUTYH*	*PRKAR1A*	*SHOX*	*TRPV3*
BCR	*DCC*	*FZD2*	*KDSR*	*MVD*	*PRKCD*	*SIX1*	*TSC1*
BDNF	*DCLRE1C*	*G6PC*	*KEAP1*	*MVK*	*PSAP*	*SIX6*	*TSC2*
BICC1	*DDB2*	*G6PC3*	*KIAA0753*	*MYC*	*PTCH1*	*SKI*	*TSG101*
BIRC3	*DDIT3*	*GABRD*	*KIF11*	*MYCL*	*PTCH2*	*SKP2*	*TSHR*
BLM	*DDX41*	*GALNT12*	*KIF1B*	*MYCN*	*PTEN*	*SLC17A9*	*TSR2*
BLNK	*DHCR24*	*GATA1*	*KIF7*	*MYD88*	*PTH1R*	*SLC22A18*	*TUBB*
BLOC1S3	*DHCR7*	*GATA2*	*KIT*	*MYH11*	*PTPN11*	*SLC25A13*	*TWIST1*
BLOC1S6	*DHH*	*GATA3*	*KITLG*	*MYH8*	*PTPN3*	*SLC26A2*	*TYR*
BMPER	*DICER1*	*GATA4*	*KLF4*	*MYLK*	*RAD1*	*SLC26A4*	*TYROBP*
BMPR1A	*DIS3L2*	*GBA*	*KLF6*	*MYO1H*	*RAD17*	*SLC37A4*	*U2AF1*
BMPR1B	*DKC1*	*GCGR*	*KLLN*	*NBEAL2*	*RAD21*	*SLC45A2*	*UBE2T*
BRAF	*DLC1*	*GCM2*	*KMT2C*	*NBN*	*RAD50*	*SLC49A4*	*UIMC1*
BRAP	*DLEC1*	*GDF2*	*KMT2D*	*NCOA3*	*RAD51*	*SLCO2A1*	*UROD*
BRCA1	*DMRT3*	*GDF5*	*KRAS*	*NCOR1*	*RAD51A*	*SLX4*	*USB1*
BRCA2	*DNAJC21*	*GDNF*	*KRIT1*	*NDP*	*RAD51B*	*SMAD2*	*USP8*
BRCC3	*DNASE1L3*	*GFI1*	*KRT1*	*NEK1*	*RAD51C*	*SMAD4*	*VAMP7*
BRE	*DNMT1*	*GFI1B*	*KRT10*	*NEK9*	*RAD51D*	*SMARCA4*	*VANGL1*
BRIP1	*DNMT3A*	*GJA1*	*KRT14*	*NELFA*	*RAD52*	*SMARCAL1*	*VANGL2*
BTK	*DOCK8*	*GJB2*	*KRT16*	*NF1*	*RAD54B*	*SMARCB1*	*VHL*
BUB1	*DPM1*	*GJB3*	*KRT17*	*NF2*	*RAD54L*	*SMARCE1*	*VPS45A*
BUB1B	*DTNBP1*	*GJB4*	*KRT5*	*NFE2L2*	*RAD9A*	*SMO*	*WAS*
BUB3	*DVL1*	*GJB6*	*KRT6B*	*NFKB1*	*RAF1*	*SNAI2*	*WASHC5*
C120rf70/FAAP100	*DVL3*	*GLI1*	*KRT9*	*NFKB2*	*RAG1*	*SOCS1*	*WDPCP*
C19ord40/FAAP24	*DYNC2H1*	*GLI3*	*L2HGDH*	*NHP2*	*RAG2*	*SOS1*	*WIPF1*
C1S	*DYNC2LI1*	*GNA11*	*LAMA3*	*NKX2-1*	*RARA*	*SOS2*	*WNT10A*
C2CD3	*ECE1*	*GNAQ*	*LAMB3*	*NLRP1*	*RASA1*	*SOX2*	*WNT5A*
CACNA1S	*ECM1*	*GNAS*	*LAMC2*	*NME1*	*RASA2*	*SOX9*	*WRAP53*
CALR	*EDN3*	*GNAS1*	*LEMD3*	*NOD2*	*RASSF1*	*SPIB*	*WRN*
CARD11	*EDNRB*	*GNB1*	*LETM1*	*NOP10*	*RB1*	*SPINK1*	*WT1*
CARD14	*EFL1*	*GNPTAB*	*LIG4*	*NOTCH1*	*RB1CC1*	*SPOP*	*WWOX*

CASP10	*EGFR*	*GPC3*	*LIN28B*	*NOTCH2*	*RBBP8/CtP*	*SPRED1*	*XIAP*
CASP8	*EIF2AK4*	*GPC4*	*LMNA*	*NOTCH3*	*RECQL*	*SPRTN*	*XPA*
CBFB	*ELA2*	*GPC6*	*LMO1*	*NPM1*	*RECQL2*	*SQSTM1*	*XPC*
CBL	*ELANE*	*GPR101*	*LMOD1*	*NR0B1*	*RECQL4*	*SRC*	*XRCC2*
CC2D2A	*ELMO2*	*GPR143*	*LMX1B*	*NR4A2*	*RERE*	*SRD5A3*	*XRCC3*
CCBE1	*ENG*	*GPR35*	*LPP*	*NR4A3*	*REST*	*SRP54*	*XRCC4*
CCDC22	*ENPP1*	*GREM1*	*LRP5*	*NR5A1*	*RET*	*SRP72*	*ZFPM2*
CCM2	*EP300*	*H19*	*LRRC8A*	*NRAS*	*RFWD3*	*SRSF2*	*ZNF350*
CCND1	*EPCAM*	*H3F3A*	*LYST*	*NRTN*	*RHBDF2*	*SRY*	
CD19	*ERBB2*	*HABP2*	*LZTR1*	*NSD1*	*RINT1*	*SSX1*	
CD27	*ERCC1*	*HACE1*	*LZTS1*	*NSD2*	*RIT1*	*SSX2*	
CD28	*ERCC2*	*HAVCR2*	*MAD2L2*	*NSD3*	*RMI1*	*STAG2*	
CD40LG	*ERCC3*	*HAX1*	*MAFA*	*NSUN2*	*RMI2*	*STAG3*	
CD70	*ERCC4*	*HBB*	*MAGT1*	*NTHL1*	*RMRP*	*STAT1*	

Supplementary Table S2

Case_no	Age at operation date (AV)	Sex	Histological diagnosis (including results from somatic sequnecing)	Soft tissue/bone/GIST/GYN	Malignant sarcoma?	Grade	Origin	Anatomy (primary tumor)
1	67	Man	GIST	GIST	GIST		Primary	Stomach
2	75	Woman	Endometrial stromal cell nodulus	GYN	N		Primary	Uterus
3	45	Man	Myxofibrosarcoma	soft tissue	Y	High	Primary	Subcutaneous
7	74	Woman	Schwannoma	GIST	N		Primary	Stomach
8	66	Man	GIST	GIST	GIST		Primary	Stomach
9	76	Man	GIST	GIST	GIST		Primary	Stomach
11	79	Woman	GIST	GIST	GIST		Primary	Stomach
12	86	Man	Leiomyosarcoma	soft tissue	Y	High	Primary	Subcutaneous
14	77	Woman	Leiomyosarcoma	GYN	Y	High	Primary	Uterus
15	53	Woman	GIST	GIST	GIST		Primary	Small intestine
16	70	Man	GIST	GIST	GIST		Primary	Stomach
18	57	Woman	Synovial sarcoma	soft tissue	Y	High	Primary	Kidney
19	74	Woman	GIST	GIST	GIST		Primary	Small intestine
20	64	Man	US	soft tissue	Y	High	Recurrence	Subcutaneous
21	68	Man	GIST	GIST	GIST		Primary	Small intestine
22	60	Woman	GIST	GIST	GIST		Primary	Stomach
23	55	Woman	Lipoma	soft tissue	N		Primary	Intramuscular
25	76	Woman	Leiomyosarcoma	soft tissue	Y	High	Primary	Retroperitoneum
26	76	Man	Leiomyosarcoma	soft tissue	Y	High	Metastasis	Intramuscular

29	76	Man	Myxofibrosarcoma	soft tissue	Y	High	Primary	Intramuscular
30	77	Man	Liposarcoma, well differentiated	soft tissue	Y	Low	Primary	Intramuscular
32	66	Woman	Leiomyosarcoma	GYN	Y	High	Primary	Uterus
35	42	Man	Schwannoma	soft tissue	N		Primary	Pelvis
36	75	Woman	Hemangioma	soft tissue	N		Primary	Subcutaneous
37	51	Woman	Tenosynovial giant cell tumor	soft tissue	N		Primary	Intraarticular
38	61	Woman	MPNST	soft tissue	Y	High	Primary	Subcutaneous
39	80	Man	Leiomyosarcoma	soft tissue	Y	High	Primary	Subcutaneous
40	55	Woman	Leiomyoma	GYN	N		Primary	Uterus
41	75	Man	Spindle cell lipoma	soft tissue	N		Primary	Subcutaneous
42	48	Man	Myolipoma	soft tissue	N		Primary	Subcutaneous
43	69	Man	Intranodal Palisaded Myofibroblastoma	soft tissue	N		Primary	Subcutaneous
44	49	Woman	Extraskeletal myxiod chondrosarcoma	soft tissue	Y	High	Primary	Subcutaneous/intramuscular
45	67	Man	Leiomyosarcoma	soft tissue	Y	High	Primary	Retroperitoneum
47	75	Man	Chordoma	bone	Y	Low	Primary	Sacrum
48	61	Man	Leiomyosarcoma	soft tissue	Y	High	Primary	Subcutaneous
49	72	Man	Hemangioma	soft tissue	N		Primary	Soft tissue
50	68	Woman	Leiomyosarcoma	GYN	Y	High	Primary	Uterus
51	65	Woman	Lipoma	soft tissue	N		Primary	Subcutaneous
52	22	Woman	Osteosarcoma, paraosteal	bone	Y	Low	Primary	Bone
53	36	Woman	Granular cell tumor	soft tissue	N		Primary	Paravertebral
54	78	Man	Spindle cell lipoma	soft tissue	N		Primary	Subcutaneous
55	35	Woman	Giant cell tumor	Bone	N		Primary	bone
56	76	Woman	GIST	GIST	GIST		Primary	Stomach
57	50	Woman	Fibromatosis	soft tissue	N		Primary	Subcutaneous
58	39	Woman	Leiomyoma	GYN	N		Primary	Uterus
59	40	Woman	Leiomyoma	GYN	N		Primary	Uterus
60	46	Man	US	soft tissue	Y	High	Primary	Intramuscular
61	74	Man	PEComa	soft tissue	Y	High	Primary	Retroperitoneum
62	62	Man	GIST	GIST	GIST		Primary	Stomach
63	70	Man	GIST	GIST	GIST		Primary	Stomach
64	58	Woman	Lipoblastoma	soft tissue	N		Primary	Subcutaneous
65	70	Man	GIST	GIST	GIST		Primary	Small intestine
66	76	Woman	Myxofibrosarcoma	soft tissue	Y	High	Primary	Subcutaneous
67	66	Man	Angiosarcoma	soft tissue	Y	High	Primary	Breast
69	50	Man	MPNST, epitheloid	soft tissue	Y	High	Primary	Soft tissue
70	55	Man	Lipoma	soft tissue	N		Primary	Intramuscular
71	49	Woman	Leiomyosarcoma	soft tissue	Y	High	Metastasis	Retroperitoneum
72	60	Woman	Pleomorphic liposarcoma	soft tissue	Y	High	Primary	Subcutaneous

73	53	Woman	Leiomyosarcoma	GYN	Y	High	Metastasis	Retroperitoneum
74	27	Man	Desmoid fibromatosis	soft tissue	N		Primary	Subcutaneous/intramuscular
75	55	Man	GIST	GIST	GIST		Primary	Stomach
76	84	Man	Pleomorphic liposarcoma	soft tissue	Y	High	Primary	Soft tissue
77	69	Woman	GIST	GIST	GIST		Primary	Stomach
78	77	Woman	US	soft tissue	Y	High	Primary	Intramuscular
79	58	Woman	Chondrosarcoma	bone	Y	High	Primary	Skeletton, axial
81	78	Man	Leiomyosarcoma	soft tissue	Y	High	Primary	Soft tissue
82	18	Woman	Osteosarcoma, chondroblastic	bone	Y	High	Primary	Pelvic bone
83	76	Man	US	soft tissue	Y	High	Primary	Subcutaneous
84	60	Man	Lipoma	soft tissue	N		Primary	Groin
85	3	Woman	Lipoblastoma	soft tissue	N		Primary	Intramuscular
86	62	Man	Liposarcoma, dedifferentiated	soft tissue	Y	Low	Primary	Retroperitoneum
87	77	Man	Schwannoma	soft tissue	N		Primary	Intramuscular
88	75	Man	MPNST	soft tissue	Y	High	Primary	Subcutaneous
89	64	Man	US	soft tissue	Y	High	Metastasis	Intramuscular
90	67	Man	US	soft tissue	Y	High	Primary	Intramscular
91	61	Man	Pleomorphic liposarcoma	soft tissue	Y	High	Primary	Subcutaneous
92	66	Woman	GIST	GIST	GIST		Primary	Stomach
93	59	Man	Pleomorphic liposarcoma	soft tissue	Y	High	Primary	Intramuscular
94	37	Woman	Fibromatosis	soft tissue	N		Primary	Intramuscular
95	72	Woman	Liposarcoma, dedifferentiated	soft tissue	Y	High	Primary	Retroperitoneum
96	78	Woman	Leiomyosarcoma	GYN	Y	High	Primary	Uterus
97	47	Man	Myxoma	soft tissue	N		Primary	Intramuscular
98	73	Man	Liposarcoma	soft tissue	Y	Low	Primary	Intramuscular
99	68	Man	Liposarcoma, dedifferentiated	soft tissue	Y	High	Primary	Retroperitoneum
100	53	Woman	Endometrial stromal cell sarcoma	GYN	Y	High	Recurrence	Uterus
101	43	Woman	Leiomyosarcoma	soft tissue	Y	High	Primary	Retroperitoneum
102	44	Man	GIST	GIST	GIST		Primary	Stomach
103	62	Woman	GIST	GIST	GIST		Primary	Stomach
104	63	Man	Myxofibrosarcoma	soft tissue	Y	High	Primary	Subcutaneous
105	58	Man	Fibromatosis	soft tissue	N		Primary	Subcutaneous
106	86	Man	US	soft tissue	Y	High	Primary	Subcutaneous
107	80	Woman	Extraskeletal myxiod chondrosarcoma	soft tissue	Y	High	Primary	Soft tissue
109	55	Woman	Elastofibroma	soft tissue	N		Primary	Subcutaneous
111	38	Woman	Leiomyosarcoma	GYN	Y	High	Metastasis	Liver
112	62	Woman	Tenosynovial giant cell tumor	soft tissue	N		Primary	Intraarticular
114	23	Man	Giant cell tumor	Bone	N		Primary	Femur

115	79	Woman	Adenosarcoma, sarcomatous overgrowth	GYN	Y	High	Primary	Uterus
116	45	Man	MPNST	Soft tissue	Y	High	Metastasis	Intramuscular
117	65	Woman	Solitary fibrous tumor	Soft tissue	Y	Low	Primary	Pleura
118	52	Woman	Solitary fibrous tumor	soft tissue	Y	Low	Primary	Subcutaneous
119	51	Woman	Phyllodes	soft tissue	Y	Low	Primary	Breast
120	56	Woman	Liposarcoma, dedifferentiated	soft tissue	Y	High	Primary	Retroperitoneum
121	26	Woman	Synovial sarcoma	soft tissue	Y	High	Primary	Soft tissue
122	65	Woman	PEComa	GYN	Y	High	Primary	Uterus
123	67	Woman	Solitary fibrous tumor	soft tissue	Y	Low	Primary	Retroperitoneum
124	70	Woman	US	soft tissue	Y	High	Primary	Subcutaneous
125	54	Woman	Leiomyosarcoma	Soft tissue	Y	High	Metastasis	Pelvis
126	75	Woman	Chondrosarcoma, dedifferentiated	soft tissue	Y	High	Primary	Soft tissue
127	77	Man	US	soft tissue	Y	High	Primary	Intramuscular
128	44	Woman	Leiomyoma	GYN	N		Primary	Uterus
129	45	Man	Leiomyosarcoma	soft tissue	Y	High	Metastasis	Perineum
130	78	Woman	Adenosarcoma, sarcomatous overgrowth	GYN	Y	High	Primary	Uterus
131	37	Woman	GIST	GIST	GIST		Primary	Stomach
132	62	Woman	Leiomyosarcoma	GYN	Y	High	Primary	Uterus
133	49	Woman	Fibromatosis	soft tissue	N		Primary	Intrabdominal
134	52	Woman	Leiomyosarcoma	GYN	Y	High	Metastasis	Uterus
135	76	Man	Liposarcoma, dedifferentiated	soft tissue	Y	High	Recurrence	soft tissue
136	57	Man	Pleomorphic liposarcoma	soft tissue	Y	High	Primary	Subcutaneous
137	63	Woman	Solitary fibrous tumor	soft tissue	Y	Low	Primary	soft tissue
139	78	Woman	Radiation induced sarcoma	soft tissue	Y	High	Metastasis	Subcutaneous
140	55	Woman	Solitary fibrous tumor	soft tissue	Y	Low	Primary	Retroperitoneum
141	53	Woman	GIST	GIST	GIST		Primary	Duodenum
142	76	Man	Leiomyosarcoma	soft tissue	Y	Low	Primary	Subcutaneous
143	71	Woman	Myxofibrosarcoma	soft tissue	Y	High	Metastasis	Intramuscular
144	82	Woman	GIST	GIST	GIST		Primary	Ileum
145	70	Man	GIST	GIST	GIST		Primary	Small intestine
146	62	Man	Liposarcoma	soft tissue	Y	Low	Primary	Retroperitoneum
147	60	Man	US	soft tissue	Y	High	Metastasis	bone
148	42	Woman	Schwannoma	soft tissue	N		Primary	soft tissue
149	53	Man	GIST	GIST	GIST		Primary	Ileum
150	74	Woman	Radiation induced sarcoma	soft tissue	Y	High	Primary	Groin
151	47	Man	GIST	GIST	GIST		Primary	Stomach

152	35	Man	MPNST	soft tissue	Y	High	Primary	Intramuscular
153	23	Man	Leiomyosarcoma	soft tissue	Y	High	Primary	soft tissue
154	49	Woman	Osteosarcoma, extraskelettal	soft tissue	Y	High	Primary	soft tissue
155	69	Man	Liposarcoma, dedifferentiated	soft tissue	Y	High	Recurrence	Intramuscular
157	79	Man	Myxofibrosarcoma	soft tissue	Y	High	Recurrence	Subcutaneous
158	79	Woman	Histiocytic sarcoma	soft tissue	Y	High	Primary	Subcutaneous
159	50	Woman	Leiomyoma	GYN	N		Primary	Uterus
160	82	Woman	MPNST	soft tissue	Y	High	Primary	Subcutaneous
161	48	Woman	Leiomyosarcoma	GYN	Y	High	Recurrence	Intrabdominal
163	36	Man	Rhabdomyosarcoma, pleomorphic	soft tissue	Y	High	Primary	soft tissue+bone
164	27	Man	Perineuroma (GAB1::ALB1)	soft tissue	N		Primary	Intramuscular
165	76	Man	GIST	GIST	GIST		Primary	Colon
166	64	Man	GIST	GIST	GIST		Primary	Stomach
167	42	Man	Myxofibrosarcoma	soft tissue	Y	High	Primary	Subcutaneous
168	58	Man	MPNST	soft tissue	Y	High	Metastasis	Subcutaneous
169	75	Man	Synovial sarcoma	soft tissue	Y	High	Primary	soft tissue
170	65	Woman	Myxofibrosarcoma	soft tissue	Y	High	Primary	Subcutaneous
171	59	Man	Chondrosarcoma	bone	Y	High	Primary	ribs/soft tissue
172	38	Woman	Phyllodes, malignant	soft tissue	Y	High	Primary	Breast
173	76	Man	US	soft tissue	Y	High	Recurrence	Subcutaneous
174	64	Man	Liposarcoma, dedifferentiated	soft tissue	Y	High	Recurrence	Intramuscular
175	79	Man	MPNST	soft tissue	Y	High	Primary	Intramuscular
176	52	Woman	Endometrial stromal cell sarcoma	GYN	Y	Low	Recurrence	Uterus
177	87	Man	Myxofibrosarcoma	soft tissue	Y	Low	Recurrence	Subcutaneous
178	74	Man	Liposarcoma, dedifferentiated	soft tissue	Y	High	Metastasis	Retroperitoneum
179	79	Man	GIST	GIST	GIST		Primary	Stomach
180	39	Woman	Leiomyosarcoma	GYN	Y	High	Metastasis	Uterus
181	82	Man	Leiomyosarcoma	soft tissue	Y	High	Primary	Subcutaneous
182	80	Man	US	soft tissue	Y	High	Metastasis	Intramuscular
183	58	Man	Chondrosarcoma, dedifferentiated	bone	Y	High	Primary	
184	82	Woman	Leiomyosarcoma	GYN	Y	High	Primary	Uterus
187	81	Man	Liposarcoma	soft tissue	Y	Low	Recurrence	Retroperitoneum
188	56	Woman	Osteosarcoma, extraskelettal	soft tissue	Y	High	Recurrence	Subcutaneous
189	67	Woman	US	soft tissue	Y	High	Primary	Intramuscular
190	73	Man	GIST	GIST	GIST		Metastasis	Colon
191	79	Woman	Myxofibrosarcoma	soft tissue	Y	High	Primary	Subcutaneous
192	54	Man	Leiomyosarcoma	soft tissue	Y	Low	Primary	Retroperitoneum
194	39	Man	LGFMS	soft tissue	Y	Low	Primary	Intramuscular

195	61	Man	Desmoplastic fibroblastoma	soft tissue	N		Primary	soft tissue
196	67	Woman	Radiation induced sarcoma	soft tissue	Y	High	Recurrence	Subcutaneous
197	54	Man	Chondrosarcoma	bone	Y	High	Primary	Pelvic bone
199	63	Man	Lipoma	soft tissue	N		Primary	Retroperitoneum
200	71	Woman	MPNST	soft tissue	Y	High	Primary	Retroperitoneum
201	43	Woman	Leiomyoma	GYN	N		Primary	Uterus
202	15	Man	Aneurysmal bone cyst	bone	N		Primary	Femur
203	57	Woman	Adenosarcoma, sarcomatous overgrowth	GYN	Y	High	Primary	Uterus
204	78	Woman	GIST	GIST	GIST		Primary	Stomach
205	55	Woman	PEComa	GYN	N		Primary	Uterus
206	79	Woman	US	soft tissue	Y	high	Primary	Subcutaneous
207	57	Man	Dermatofibrosarcoma protuberans, fibrosarcoma	soft tissue	Y	low	Primary	Subcutaneous
208	49	Man	Leiomyosarcoma	soft tissue	Y	High	Recurrence	Intrabdominal
209	78	Woman	Solitary fibrous tumor, malignant	soft tissue	Y	High	Recurrence	Subcutaneous
210	69	Man	US	soft tissue	Y	High	Primary	Intramuscular
211	46	Woman	Myxoid liposarcoma	soft tissue	Y	Low	Metastasis	Intramuscular
212	66	Woman	Leiomyoma	GYN	N		Primary	Uterus
213	64	Woman	Osteosarcoma, chondroblastic	bone	Y	High	Metastasis	bone
214	61	Woman	Elastofibroma	Soft tissue	N		Primary	Subcutaneous
215	23	Woman	Alveolar soft part sarcoma	Soft tissue	Y	High	Primary	Intramuscular
216	61	Man	Liposarcoma, dedifferentiated	Soft tissue	Y	High	Primary	Small intestine
217	54	Woman	GIST	GIST	GIST	High	Primary	Stomach
218	44	Woman	Leiomyoma	GYN	N		Primary	Uterus
220	67	Man	Leiomyosarcoma	Soft tissue	Y	High	Primary	Retroperitoneum
221	82	Man	GIST	GIST	GIST		Primary	Small intestine
222	76	Woman	MPNST	Soft tissue	Y	High	Recurrence	
223	39	Man	GIST	GIST	GIST		Primary	Small intestine
226	65	Man	Myxoid liposarcoma	Soft tissue	Y	Low	Primary	Subcutaneous
227	54	Man	Dermatofibrosarcoma protuberans	Soft tissue	Y	Low	Primary	Subcutaneous
228	49	Man	GIST	GIST	GIST		Primary	Stomach
229	82	Man	Myxofibrosarcoma	Soft tissue	Y	High	Recurrance	
230	78	Woman	Neurofibroma / Schwannoma hybrid nerve sheath tumor	Soft tissue	N		Primary	Soft tissue
231	52	Man	Liposarcoma dedifferentiated	Soft tissue	Y	High	Primary	Soft tissue
232	63	Woman	Myxofibrosarcoma	Soft tissue	Y	High	Primary	Subcutaneous
233	84	Woman	Histiocytic sarcoma	Soft tissue	Y	High	Primary	Subcutaneous

234	66	Woman	Solitary fibrous tumor	Soft tissue	Y	Low	Primary	Pleura
235	15	Woman	Chondrosarcoma	Bone	N		Primary	Bone
236	73	Woman	Leiomyosarcoma	GYN	Y	High	Primary	Uterus
237	47	Man	Myoepithelial carcinoma	Soft tissue	Y	High	Primary	Soft tissue
238	61	Woman	Solitary fibrous tumor	Soft tissue	Y	High	Primary	RIbs
240	48	Man	Dermatofibrosarcoma protuberans	Soft tissue	Y	Low	Recurrance	Subcutaneous
241	77	Woman	Myxofibrosarcoma	Soft tissue	Y	High	Primary	Intramuscular
242	76	Man	Myxoid sarcoma	Soft tissue	Y	Low	Primary	Lung
243	56	Man	Myxofibrosarcoma	Soft tissue	Y	High	primary	Intramuscular
244	24	Woman	Giant cell tumor	Bone	N		Primary	bone
246	43	Man	Schwannoma	Soft tissue	N		Primary	Soft tissue
247	28	Woman	Myopericytomatosis	Soft tissue	N		Primary	Intramuscular
248	48	Woman	Phosphaturic mesenchymal tumor	Soft tissue	N		Primary	Subcutaneous
249	61	Woman	Myoepithelial carcinoma	Soft tissue	Y	High	Recurrance	Subcutaneous
250	49	Woman	Leiomyosarcoma	GYN	Y	High	Primary	Uterus
251	78	Man	Extra skeletal myxoid condrosarcoma	Soft tissue	Y	High	Primary	Subcutaneous
252	66	Woman	Liposarcoma well differentiated	Soft tissue	Y	Low	Primary	Retroperitoneum
253	64	Woman	Sarcoma	Soft tissue	Y	High	Metastasis	-
254	60	Man	Extraskeletal osteosarcoma	Soft tissue	Y	High	Recurrance	Subcutaneous
256	38	Man	GIST	GIST	GIST	High	Primary	Small intestine
257	70	Man	Chondrosarcoma	Bone	Y	High	Primary	Bone
258	56	Woman	Lipoblastomatosis	Soft tissue	Y	Low	Recurrance	Retroperitoneum
259	42	Man	GIST	GIST	GIST		Primary	Small intestine
260	56	Woman	Leiomyoma	GYN	N		Primary	Uterus
261	76	Woman	Leiomyosarcoma	Soft tissue	Y	High	Primary	Retroperitoneum
262	87	Woman	Myxofibrosarcoma	Soft tissue	Y	High	Primary	Intramuscular
263	36	Woman	Extraskeletal myxoid chondrosarcoma	soft tissue	Y	High	Primary	Intramuscular
265	45	Woman	Leiomyoma	GYN	N		Primary	Uterus
266	74	Woman	GIST	GIST	GIST	Low	Primary	Stomach
267	64	Woman	Angiosarcoma	Soft tissue	Y	High	Primary	breast
268	72	Woman	Mesonephric-like carcinosarcoma	GYN	N		Primary	Uterus
269	36	Woman	Scwhannoma	Soft tissue	N		Recurrance	Soft tissue
270	58	Man	Chondrosarcoma	Bone	Y	High	Primary	Bone
271	73	Man	Leiomyosarcoma	Soft tissue	Y	High	Primary	Retroperitoneum
272	42	Man	Fibrohistiocytoma	Soft tissue	N		Primary	SUbcutanous
273	20	Woman	Osteosarcoma, dedifferentiated	Soft tissue	Y	High	Recurrance	Subcutaneous

274	62	Man	US	Soft tissue	Y	High	Metastasis	Soft tissue
275	24	Woman	Giant cell tumor of soft-parts	Soft tissue	N		Primary	Subcutaneous
276	58	Woman	Liposarcoma	Soft tissue	Y	High	Recurrance	Retroperitoneum
277	60	Man	Myxofibrosarcoma	Soft tissue	Y	High	Primary	Subcutaneous
278	59	Woman	GIST	GIST	GIST		Primary	Stomach
279	30	Woman	Epitheloid sarcoma	Soft tissue	Y	High	Recurrance	Soft tissue
280	69	Woman	Leiomyosarcoma	Soft tissue	Y	High	Primary	Subcutaneous
281	23	Woman	Desmoid fibromatosis	Soft tissue	Y		Primary	Small intestine
282	55	Woman	US	Soft tissue	Y	High	Primary	Subcutaneous
284	65	Man	Atypical spindle cell lipoma	Soft tissue	N		Primary	Subcutaneous
285	61	Man	GIST	GIST	GIST		Primary	Stomach
286	62	Woman	Solitary fibrous tumor	Soft tissue	Y	Low	Primary	Subcutaneous
287	13	Woman	RAF1-associated sarcoma	Soft tissue	Y		Primary	Intramuscular
288	76	Woman	Osteosarcoma	Bone	Y	High	Primary	Femur
290	52	Man	Chondrosarcoma, grade 1	Bone	Y	Low	Primary	Rib
291	45	Woman	Solitary fibrous tumor	Soft tissue	Y	Low	Primary	Intraabdominal
292	63	Woman	Leiomyosarcoma	Soft tissue	Y	High	Metastasis	Uterus
293	74	Woman	Malign OFMT	Soft tissue	Y	High	Primary	Intraarticular
295	38	Woman	Secondary peripheral chondrosarcoma	Bone	Y	Low	Primary	Femur
297	46	Man	Metastatic osteosarcoma	Bone	Y	High	Metastasis	mandible
298	34	Man	High-grade sarcoma with SRF::E2F1 fusion	Soft tissue	Y	High	Primary	Subcutaneous
299	75	Man	Pleomorfic sarcoma	Soft tissue	Y	High	Primary	Intramuscular
300	48	Man	Epitheloid sarcoma	Soft tissue	Y	High	Primary	Intramuscular
301	82	Man	Leiomyosarcoma	Soft tissue	Y	High	Recurrance	Subcutaneous
302	58	Man	Myxofibrosarcoma	Soft tissue	Y	High	Primary	Intramuscular
304	80	Woman	Leiomyosarcoma	Soft tissue	Y	High	Primary	Retroperitoneum
306	25	Man	MPNST	Soft tissue	Y	High	Metastasis	Brain
307	43	Woman	Leiomyoma	GYN	N		Primary	Uterus
308	71	Man	US	Soft tissue	Y	High	Primary	Subcutaneous
309	72	Man	Myxofibrosarcoma	Soft tissue	Y	High	Primary	Subcutaneous
311	73	Woman	Synovial chondromatosis	Soft tissue	N		Primary	Soft tissue
312	59	Man	Liposarcoma	Soft tissue	Y	Low	Primary	Retroperitoneum
313	65	Woman	GIST	GIST	GIST		Primary	Small intestine
314	46	Woman	Leiomyoma	GYN	N		Primary	Uterus
315	69	Man	Myxofibrosarcoma	Soft tissue	Y	High	Primary	Subcutaneous

317	21	Woman	Ewing sarcoma	Bone	Y	High	Primary	bone
318	36	Man	Myxoinflammatory fibroblastic sarcoma	Soft tissue	Y	Low	Primary	Subcutaneous
319	61	Woman	Liposarcoma, dedifferentiated	Soft tissue	Y	High	Primary	Intramuscular
320	11	Man	Chondroblastic osteosarcoma	Bone	Y	High	Primary	Femur
321	42	Woman	Leiomyosarcoma	GYN	Y	High	Recurrance	Intraabdominal
322	76	Woman	atypical cartilagenous tumor	Bone	Y	Low	Primary	Femur
323	82	Man	MPNST	Soft tissue	Y	High	Primary	Intramuscular
324	39	Woman	Synovial sarcoma	Soft tissue	Y	High	Primary	Stomach
325	38	Man	Chondrosarcoma, G1	Bone	Y	Low	Primary	Ilium
326	31	Woman	Low grade paraosteal osteosarcoma	Bone	Y	Low	Primary	Humerus
328	54	Woman	Leiomyoma (kontrollera)	GYN	N		Primary	Uterus
329	48	Man	Low-grade paraosteal OS	Bone	Y	Low	Primary	Bone
330	46	Woman	Leiomyosarcoma	GYN	Y	High	Metastasis	Uterus
331	63	Man	Liposarcoma	Soft tissue	Y	High	Primary	Intramuscular
335	77	Woman	US	Soft tissue	Y	High	Primary	Intramuscular
336	73	Woman	Osteosarcoma, fibroblastic	Bone	Y	High	Primary	Soft tissue/bone
337	81	Man	Myxofibrosarcoma	Soft tissue	Y	High	Primary	Soft tissue
338	37	Woman	Liposarcoma	Soft tissue	Y	Low	Primary	Retroperitoneum
339	70	Man	Complete response (clinical diagnosis soft tissue sarcoma paravertebral)	Soft tissue	Y	High	Primary	Paravertebral
340	77	Woman	Liposarcoma dedifferentiated	soft tissue	Y	high	Primary	Retroperitoneum
343	80	Woman	Angiosarcoma	soft tissue	Y	High	Recurrance	Subcutaneous
344	60	Woman	Lipoblastoma	GYN	N	-	Primary	Uterus
345	84	Man	US	Soft tissue	Y	High	Recurrance	Subcutaneous
346	82	Woman	Spindle cell sarcoma	Soft tissue	Y	Low	Primary	Intramuscular
347	78	Woman	US	Soft tissue	Y	High	Primary	Subfascial
348	56	Man	Atypical spindle/pleomorphic lipomatous tumor	Soft tissue	Y	Low	Primary	Subcutaneous
349	79	Man	Malignant bone tumor	Bone	Y	-	Primary	Periosteal
352	84	Man	Spindle sarcoma low grade	Soft tissue	Y	Low	Primary	Paraspinal
353	59	Woman	Leiomyosarcoma	soft tissue	Y	High	Primary	Uterus
354	68	Man	Chondrosarcoma	Bone	Y	Low	Primary	Bone
355	43	Man	Desmoid fibromatosis	Soft tissue	N	-	Primary	Intramuscular
356	22	Man	Alveolar soft part sarcoma	Soft tissue	Y	High	Primary	Intramuscular

360	87	Woman	Fibroblastic sarcoma	Soft tissue	Y	Low	Primary	Subcutaneous
361	29	Man	Myoepithelial tumor	Soft tissue/bone	N	-	Primary	Soft tissue/bone
362	67	Woman	Myxofibrosarcoma	Soft tissue	Y	High	Primary	Subcutaneous
364	28	Woman	GIST	GIST	GIST		Primary	Stomach

GIST: Gastronintestinal stromal tumor, GYN: gynaecological tumor, MPNST: malignant peripheral nerve sheath tumor, US: undifferentiated sarcoma, N: no, Y: yes

Supplementary Table S3

**Clinical characteristics**	**No germline finding***	**Germline finding****	**Significance**
Age at diagnosis [mean years]	62	58	P=0.4 (NS)
Sex [% women]	53	54	Odds ratio 1.1 (NS)
Malignant sarcoma [%]	67	71	Odds ratio 1.2 (NS)

Supplementary Table S4

Supplementary table S4 Non-tumor syndrome participants

**Histological diagnosis after sequencing**	**Germline P/LP variant**	**Clinical impact**	**Second hit summary**
GIST	GJB2, NM_004004, c.35del, p.Gly12ValfsTer2	Risk factor	No second hit (compound germline)
GIST	POLG,ENST00000268124.5:c.2890C>T,R964C	Risk factor	Deletion 15q
Fibromatosis	KCNQ1, NM_181798, c.573_577del, p.Arg192CysfsTer91	Clinical action	SNV: KCNQ1,NM_181798,c.284G>A,p.Ser95Asn
Liposarcoma, dedifferentiated	SPINK1, NM_003122, c.194+2T>C	Risk factor	No second hit
Osteosarcoma, extraskelettal	GJB2, NM_004004, c.35del, p.Gly12ValfsTer2	Risk factor	No second hit (compund germline)
Liposarcoma, dedifferentiated	TGFBR2, NM_001024847, c.1685G>A, p.Arg562His	Clinical action	No second hit
Leiomyoma	TNFRSF13B, NM_012452, c.260T>A, p.Ile87Asn	Risk factor	No second hit
Aneurysmal bone cyst	IKBKG, NM_003639, c.1219A>G,p.Met407Val	Risk factor	No second hit
GIST	MPL, NM_005373, c.305G>C, p.Arg102Pro	Risk factor	No second hit
PEComa	TNFRSF13B, NM_012452, c.310T>Cp.Cys104Arg	Risk factor	No second hit
Undifferentiated pleomorphic sarcoma	KCNQ1, NM_181798, c.1552C>T, p.Arg518Ter	Clinical action	No second hit
Myxofibrosarcoma	KCNQ1, NM_181798, c.914G>A, p.Trp305Ter	Clinical action	No second hit

GIST: gastrointestinal stromal tumor

Supplementary Figure S1

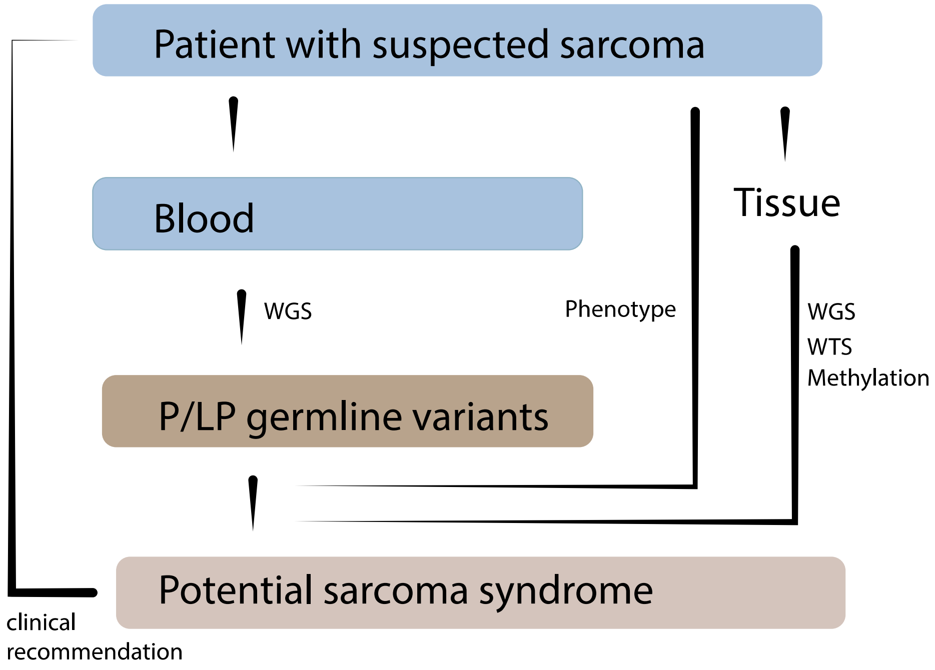

WGS: whole genome sequencing, WTS: whole transcriptome sequencing
